# An Extremely Highly Sensitive ELISA in pg mL^−1^ Level Based on a Newly Produced Monoclonal Antibody for the Detection of Ochratoxin A in Food Samples

**DOI:** 10.3390/molecules28155743

**Published:** 2023-07-29

**Authors:** Yexuan Ren, Ruwen Tian, Ting Wang, Junlin Cao, Jianguo Li, Anping Deng

**Affiliations:** The Key Lab of Health Chemistry and Molecular Diagnosis of Suzhou, College of Chemistry, Chemical Engineering and Materials Science, Soochow University, Renai Road 199, Suzhou 215123, China; renyexuan8@163.com (Y.R.); ruwent@163.com (R.T.); m13383897519@163.com (T.W.); cjl9808@163.com (J.C.)

**Keywords:** enzyme-linked immunosorbent assay (ELISA), monoclonal antibody (mAb), mycotoxin, ochratoxin A (OTA), food samples, sensitive detection

## Abstract

In this study, an extremely highly sensitive enzyme-linked immunosorbent assay (ELISA) based on a newly produced monoclonal antibody (mAb) for the detection of ochratoxin A (OTA) in food samples was developed. OTA-Bovine serum albumin (BSA) conjugate was prepared and used as the immunogen for the production of the mAb. Among four hybridoma clones (8B10, 5C2, 9B7, and 5E11), the antibody from 8B10 displayed the highest affinity recognition for OTA. Based on the mAb (8B10), the IC_50_ and LOD of the ELISA for OTA were 34.8 pg mL^−1^ and 1.5 pg mL^−1^, respectively, which was 1.53~147 times lower than those in published ELISAs, indicating the ultra-sensitivity of our assay. There was no cross-reactivity of the mAb with the other four mycotoxins (AFB_1_, ZEN, DON, and T-2). Due to the high similarity in molecular structures among OTA, ochratoxin B (OTB), and ochratoxin C (OTC), the CR values of the mAb with OTB and OTC were 96.67% and 22.02%, respectively. Taking this advantage, the ELISA may be able to evaluate total ochratoxin levels in food samples. The recoveries of the ELISA for OTA in spiked samples (corn, wheat, and feed) were 96.5–110.8%, 89.5–94.4%, and 91.8–113.3%; and the RSDs were 5.2–13.6%, 8.2–13.0%, and 7.7–13.7% (*n* = 3), respectively. The spiked food samples (corn) were measured by ELISA and HPLC-FLD simultaneously. A good correlation between ELISA (x) and HPLC-FLD (y) with the linear regression equation of y = 0.918x − 0.034 (R^2^ = 0.985, *n* = 5) was obtained. These results demonstrated that the newly produced mAb-based ELISA was a feasible and ultra-sensitive analytical method for the detection of OTA in food samples.

## 1. Introduction

Ochratoxin A (OTA) is a ubiquitous secondary metabolite of several Aspergillus and Penicillium species that occurs in different agricultural commodities during agricultural production and storage [1,2]. It has been widely detected in foodstuff and beverages such as cereals, cereal-derived products, wheat, corn, peanuts, dried fruits, spices, chili, coffee, grape juice, beer, and wine [3,4]. It is also present in animal products, such as meat and dairy products, where the toxin can be transferred and accumulated when the animals ingest contaminated feed [5,6]. OTA has been attracting worldwide attention because it presents a significant risk to human and animal health and causes economic losses. Toxicological studies indicate that OTA exhibits strong toxic activities such as nephrotoxicity, hepatotoxicity, teratogenicity, carcinogenicity, mutagenicity, neurotoxicity, and immunotoxicity [7,8,9]. To ensure food safety, the European Commission has established strict maximum-level limits for OTA in raw cereal grains and roasted coffee (5 μg/kg), in cereals and cereal products intended for human consumption (3.0 μg/kg), in wine and grape juice (2 μg/kg), and in baby food and cereal-based food intended for young children (0.5 μg/kg) [10]. Therefore, the development of highly sensitive and specific analytical methods for the detection of OTA in food samples is urgently required.

The typical analytical methods used for the detection of OTA in food samples are mainly based on HPLC with a fluorescence detector (FLD) [11,12,13] or liquid chromatography/tandem mass spectrometry (LC-MS/MS) [14,15,16]. Although chromatographic methods are sensitive and accurate, they are very expensive, with a high testing cost, low throughput, and are time consuming, as they require skilled operators, complex sample pretreatment, and expensive instruments, which are unsuitable for routine screening in food safety monitoring.

Immunoassays, especially enzyme-linked immunosorbent assays (ELISAs), are analytical methods that are based on the specific interaction between an antibody and the corresponding antigen. Immunoassays are generally rapid and have high sensitivity and specificity, simple sample preparation, high throughput, and, therefore, low cost for each sample [17,18,19]. In the last decades, many immunoassays using different signal readouts, such as chemiluminescence [20], time-resolved fluorescence [21], electrochemical [22], etc., have reported the detection of OTA. Also, many polyclonal antibody (pAb)- or monoclonal antibody (mAb)-based indirect competitive ELISAs (ic-ELISA) and direct competitive ELISAs (dc-ELISA) have been developed for the detection OTA in food samples (Table 1) [23,24,25,26,27,28,29,30,31,32,33,34,35,36]. Usually, for the developed immunoassays, the first purpose is to achieve the high sensitivity of the assay for the target analyte. However, the fundamental factor to determine the sensitivity of the immunoassay is mainly dependent upon the quality of the antibody. The production of a high-quality antibody, with the properties of high sensitivity and specificity for the target analyte, is always the main goal for immuno-analytical scientists to pursue. 

In this study, after making great efforts, we have successfully produced a mAb with the highest affinity binding force for OTA by using hybridoma technology. Based on this mAb, the corresponding ic-ELISA for OTA was established with IC_50_ (e.g., the concentration of OTA producing 50% of the signal inhibition in the ELISA standard curve) and limit of detection (LOD) values of 34.8 pg mL^−1^ and 1.5 pg mL^−1^, respectively, which are about 1.53~147 times lower than those obtained in other ELISAs. To the best of our knowledge, this is the most sensitive pg mL^−1^ level in ELISA achieved so far based on this newly produced mAb. In addition, OTA-spiked samples (corn, wheat, and feed) were measured by the proposed ELISA. The results from the spiked food (corn) samples were confirmed by HPLC-FLD.

## 2. Results and Discussion

### 2.1. Determination of Coupling Ratio in OTA–Protein Conjugates

**Figure 1:**
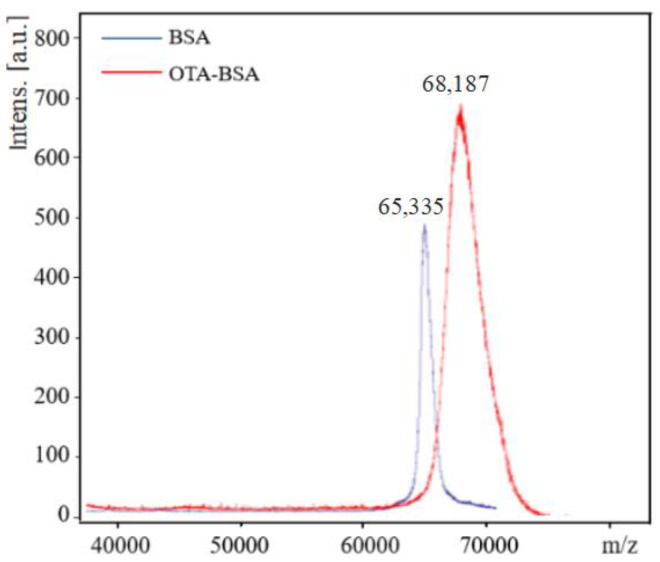
MALDI-TOF-MS spectra of BSA and OTA–BSA. The *m*/*z* values of BSA and OTA–BSA are 65,335 and 68,187, respectively.

OTA was coupled to BSA and OVA via the carboxylic group by the carbodiimide method to form two conjugates, e.g., OTA–BSA and OTA–OVA. The coupling ratio of OTA/protein in the conjugates was determined by MALDI-TOF-MS. The typical MALDI-TOF-MS spectra of BSA and OTA–BSA conjugates are illustrated in Figure 1. It can be seen from Figure 1 that the molecular weights of BSA and OTA–BSA are 65,335 and 68,187, respectively. As the molecular weight of OTA is 403, the coupling ratio of OTA/BSA in the OTA–BSA conjugate is estimated to be (68,187 − 65,335)/403 = 7.1, which indicates that in each BSA molecule, about 7.1 OTA molecules have been linked to surface of BSA. The MALDI-TOF-MS spectra of OVA and OTA–OVA are similar to those in Figure 1 and are not given herein. Similar calculation results show that the coupling ratio of the OTA–OVA conjugate is 5.4. These results indicate that the OTA has successfully coupled to the carrier proteins.

### 2.2. Production of mAb against OTA 

Five female mice were immunized subcutaneously with OTA–BSA as the immunogen. After the sixth immunization, the sera from the immunized mice were collected and tested by ic-ELISA. Among the five immunized mice, the serum from mouse No. 1 showed the highest titer and inhibition effect tested by ic-ELISA, therefore, the spleen cells from that mouse were collected for cell fusion. 

**Figure 2:**
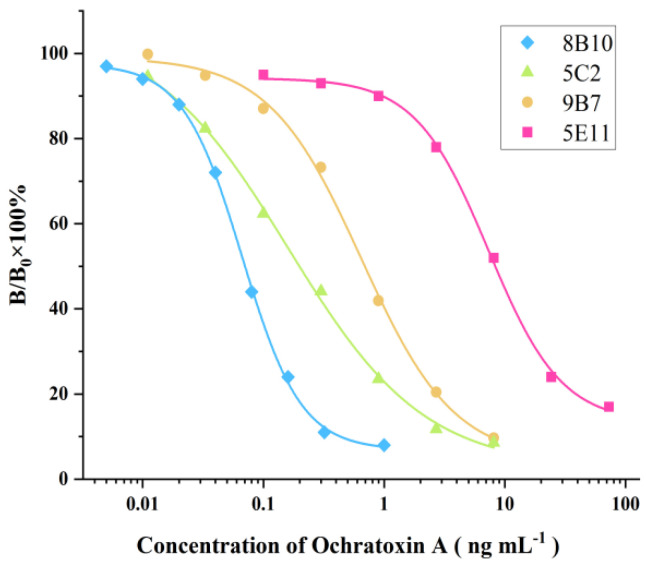
ELISA standard curves for OTA detection based on supernatants of 8B10, 5C2, 9B7, and 5E11. IC_50_ values were 0.199, 8.34, 0.693, and 0.0704 ng mL^−1^, respectively.

Ten days later, four positive wells were found by ic-ELISA using OTA as the competitor. It was found that these four hybridoma clones, named 8B10, 5C2, 9B7, and 5E11, were able to stably secrete highly sensitive antibodies against OTA. The corresponding ic-ELISA standard curves are shown in Figure 2. It was found the IC_50_ values of the ic-ELISAs based on 8B10, 5C2, 9B7, and 5E11 were within 0.1~10 ng mL^−1^. Obviously, the ic-ELISA using 8B10 displayed the highest sensitivity for OTA, thus, 8B10 was selected for subcloning via the limiting dilution method. The mAb-producing clones (8B10) were expended and cryopreserved in liquid nitrogen. The large-scale production of mAb was from nude mice ascites. 8B10 hybridoma cells were intraperitoneally injected into nude mice that were pre-injected with liquid paraffin 1 week before. Seven days after the injection, the ascites from the nude mice were collected and purified by the protein G immuno-affinity extraction technique. The purified mAb was stored at −20 °C in the presence of 50% glycerol.

### 2.3. Optimization of ELISA Conditions

In order to obtain high sensitivity, the assay conditions, including the dilution of the coating antigen, the dilution of the antibody, and GaMIgG-HRP, should be carefully optimized. The optimization was performed according to two criteria, i.e., (1) to obtain an IC_50_ value as low as possible; and (2) to obtain an absorbance value in the range of 0.8~1.5 for the zero standard concentration according to the Lambert–Beer law.

The checkerboard titration method was used to optimize the assay conditions. The coating antigen (OTA–OVA) was diluted in the range of 1:1000~1:10,000, while the dilutions of the mAb (8B10) and GaMIgG-HRP were tested in the range of 1:10,000~1:80,000 and 1:1000~1:20,000, respectively. The final optimal conditions of the ic-ELISA for OTA detection were as follows: the coating antigen was diluted at 1:2000, the mAb was diluted at 1:60,000, and GaMIgG-HRP was diluted at 1:5000. 

### 2.4. Sensitivity of the ELISA for OTA

**Figure 3:**
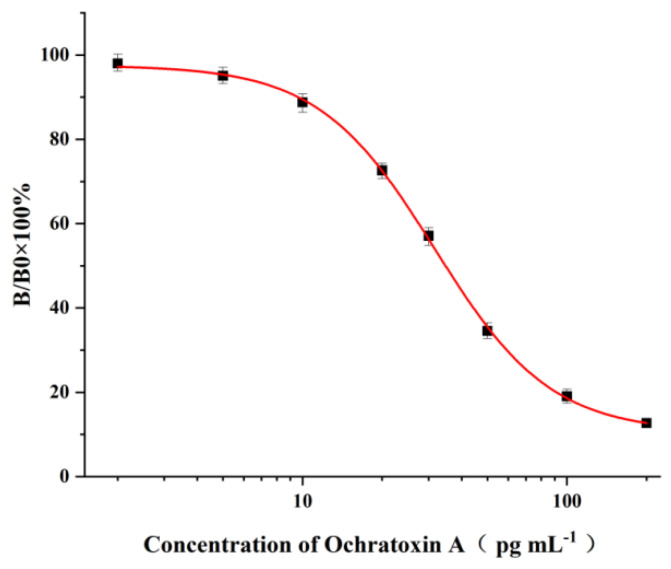
Standard curve of the ELISA for OTA detection under optimal experimental conditions. The bars represent the standard derivations of the values of B/B_0_ × 100% (*n* = 6). The IC_50_ was 34.8 pg mL^−1^, and the LOD was 1.5 pg mL^−1^.

Under the optimal conditions, the OTA standard solutions at the concentration range of 2~200 pg mL^−1^ were applied to the ic-ELISA procedures, and the corresponding standard curve is illustrated in Figure 3. It can be seen from Figure 3 that the average IC_50_ value calculated from standard curves obtained on six consecutive days was 34.8 pg mL^−1^, while the limit of detection (LOD) of the ELISA based on three times the standard deviation (3 × SD) at zero concentration was found to be 1.5 pg mL^−1^. 

In the last decades, many ELISAs for the detection of OTA in food samples based on pAb or mAb have been developed [23,24,25,26,27,28,29,30,31,32,33,34,35,36]. As shown in Table 1, the IC_50_ (or LOD) value achieved in our ELISA is about 1.53~147 times lower than those obtained in other ELISAs, which clearly demonstrates the high sensitivity of our ELISA. To the best of our knowledge, this is the most sensitive ELISA that has been achieved so far based on this newly produced mAb. In should be addressed that some aptamer (or Phage)-based ELISAs or sensors may obtain high sensitivity for OTA detection [37,38,39,40].

The high sensitivity of the newly produced mAb may be related to the coupling ratio in the immunogen (OTA–BSA) and in the coating antigen (OTA–OVA). An appropriate coupling ratio (7.1) of OTA/BSA in the immunogen may be beneficial to promote a strong immune response in immunized mice, and thus produce a high-quality antibody with high affinity recognition for the target analyte (OTA). On the other hand, the relatively lower coupling ratio (5.4) of OTA/OVA in the coating antigen may lead to a lower affinity binding force between the antibody and the coating antigen, which comparatively increases the affinity binding force between the antibody and OTA, thus enhancing the sensitivity further.

### 2.5. Specificity of the ELISA

The specificity of ELISA was evaluated by the cross-reactivity (CR) of ELISA with two OTA-structure-related compounds (OTB and OTC) and four kinds of other mycotoxins (AFB_1_, ZEN, DON, and T-2). The CR values of the assay with these compounds are presented in Table 2. When the CR of the ELISA of OTA is considered to be 100%, it can be seen from Table 2 that there is no cross-reactivity (CR < 0.01%) of the mAb with AFB_1_, ZEN, DON, or T-2, indicating that the existence of other mycotoxins in food samples will not interfere with the detection of OTA measured by the proposed ELISA. However, the CR values of the mAb with OTB and OTC were found to be 96.67% and 22.02%, respectively. The high CR values of the mAb with OTB and OTC are mainly due to the high similarity of the molecular structures among OTA, OTB, and OTC. In fact, the high CR values of the mAb with OTB and OTC can be an advantage to evaluate all of the ochratoxins (OTA, OTB, and OTC) in food samples; however, the toxicity of OTB and OTC is lower than that of OTA. To accurately detect OTA, OTB, and OTC in food samples, further research will be focused on the preparation an immuno-affinity column (in which the mAbs are immobilized on the supporter particles), which is used for the extraction of OTA, OTB, and OTC from food samples, thereafter the extracts are analyzed by LC-MS/MS. 

### 2.6. Sample Spiking and Extraction

In this study, due to the ultra-sensitivity of the proposed ELISA, it was possible to sacrifice some degree of sensitivity of the assay and to adopt a simple sample pretreatment (e.g., extracting the spiked or real food sample, diluting the extracts, and then applying the diluted extracts in the ELISA procedure). In order to completely eliminate the matrix effect and solvent effect, the appropriate dilution times of the extracts should be investigated. A total of 20 g of food samples (wheat, corn, and feed), free from OTA, were extracted with 40 mL of 60% methanol. The samples were vortexed for 10 min and centrifuged at 2000 rpm for 15 min. The supernatant was diluted at 1:5, 1:10, 1:20, 1:50, 1:100, and 1:200 with PBS, and then the diluted solutions were used to prepare the OTA standards and applied to the ELISA procedure. It was found that the IC_50_ value of the ELISA using 1:20 dilution was almost the same as the IC_50_ value of the ELISA using pure PBS, which clearly demonstrated that, at 1:20 dilution, the matrix effect and the solvent effect were completely eliminated. 

The accuracy and precision of the ELISA were verified by a spiking experiment, in which standard OTA was added to wheat, corn, and feed samples at spiking levels ranging from 1 to 100 ng g^−1^. After extraction, depending on the concentration spiked, the extracts were diluted 30~3000 times with PBS and then applied directly to the ELISA procedure. The recovery and coefficient of variation results of the ELISA for OTA are given in Table 3. The recoveries of the ELISA for OTA in the spiked samples were 96.5–110.8%, 89.5–94.4%, and 91.8–113.3%, respectively; and the corresponding RSDs were 5.2–13.6%, 8.2–13.0%, and 7.7–13.7% (*n* = 3), respectively. In addition, the inter-assay RSD was 5.4–12.8% (*n* = 3). 

### 2.7. Validation of the ELISA with HPLC-FLD

**Figure 4:**
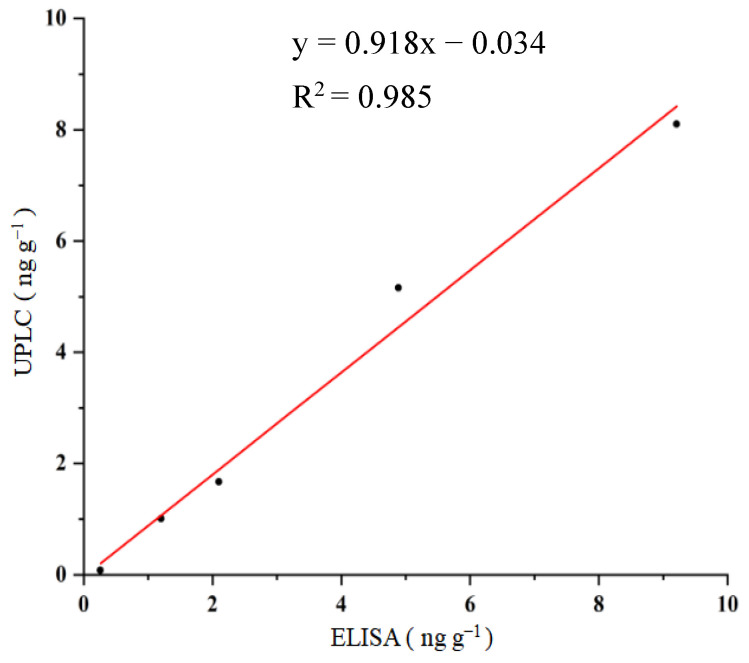
Correlation between ELISA and HPLC for OTA detection.

Under HPLC-FLD experimental conditions, the retention time of OTA was 1.46 min. When the OTA standards (0, 0.2, 1, 5, 10, and 20 ng mL^−1^) were subjected to HPLC-FLD, the corresponding signals were obtained, and the standard curve of HPLC-FLD for OTA with linear regression equation of: y = 83,947x + 1395.5 (R^2^ = 0.999, *n* = 6) was achieved. To validate the proposed ELISA, food samples (using corn as an example) were spiked with OTA at contents of 0, 1, 2, 5, and 10 ng g^−1^. After sample pre-treatment, the extracts were applied to the procedures of the ELISA and HPLC-FLD. The comparison of the ELISA with the HPLC-FLD is shown in Figure 4. A good correlation between the ELISA (x) and HPLC (y), with the linear regression equation of y = 0.918x−0.034 (R^2^ = 0.985, *n* = 5), was obtained. These results suggested that OTA in food samples could be rapidly and accurately detected by the proposed ELISA.

## 3. Materials and Methods

### 3.1. Reagents, Materials, and Apparatus

OTA and other compounds used to test for cross-reactivity (CR), including ochratoxin B (OTB), ochratoxin C (OTC), aflatoxin B_1_ (AFB_1_), zearalenone (ZEN), deoxynivalenol (DON), and trichothecenes (T-2) (Table 2), were purchased from Aladdin (Shanghai, China). Bovine serum albumin (BSA), ovalbumin (OVA), casein, RMI-1640 medium, dimethylformamide (DMF), anhydrous tetrahydrofuran (THF), 3,3,5,5-tetramethylbenzidine (TMB), N-hydroxysuccinimide (NHS), polyethylene glycol (PEG4000), N,N′-dicyclohexyl carbodiimide (DCC), and Freund’s complete and incomplete adjuvants were purchased from Sigma (St. Louis, MI, USA). Hypoxanthine aminopterin thymidine (HAT) and hypoxanthine thymidine (HT) were purchased from Gbico (Carlsbad, CA, USA). Horseradish peroxidase-conjugated goat anti-mouse IgG conjugate (HRP-GaMIgG) was purchased from Zhongshan Jinqiao Biotechnology Co., Ltd. (Beijing, China). Fetal bovine serum (FBS) was purchased from Life Technologies (Grand Island, NY, USA). Mouse SP2/0 myeloma cells were purchased from the Cell Bank of the Chinese Academy of Sciences (Shanghai, China). BALB/C mice (6–8 weeks old) were obtained from the School of Medicine, Soochow University (Suzhou, China). All other chemicals and organic solvents were of analytical grade.

The cell culture plates (24 and 96 Wells) and cell culture flasks were purchased from Corning (Corning, NY, USA). The CO_2_ incubator (HF 151 UV), freeze dryer (Alpha 1–2 LDplus), and Ultra-clean table were purchased from Heal Force Development Co., Ltd. (Hong Kong, China), Wifen Electronic Technology Co., Ltd. (Tokyo, Japan), and Haier Group (Qingdao, China), respectively. The ELISA reader (Sunrise Remote/Touch Screen) and microtiter washing machine (M12/2R) were purchased from Columbus Plus (Tecan, Grödig, Austria). The microtiter plate shaker (KJ-201C Oscillator) was bought from Kangjian Medical Apparatus, Co., Ltd. (Jiangsu, China). The deionized reverse osmosis water supply system (Dura 12FV) was purchased from The LAB Com. (Dover, DE, USA). The MALDI-TOF-MS equipment (Micro Q-TOF) was purchased from Bruker Daltonics (Billerica, MA, USA).

### 3.2. Buffers and Solutions

The buffers and solutions used in this study were as follows: (1) coating buffer was 0.05 mol L^−1^ carbonate buffer (pH 9.6); (2) coating antigen stock solution (1.0 mg mL^−1^) was prepared by dissolving 1.0 mg of OTA–OVA conjugate in 1.0 mL of coating buffer; (3) assay buffer was 0.01 mol L^−1^ phosphate buffered saline (PBS, pH 7.4); (4) washing buffer (PBST) was an assay buffer containing 0.1% (*v*/*v*) of Tween-20; (5) OTA stock solution (1.0 mg mL^−1^) was prepared by dissolving 1.0 mg of OTA in 1.0 mL of methyl alcohol; (6) OTA working solution (1000 ng mL^−1^) was prepared by diluting OTA stock solution (1.0 mg mL^−1^) with an assay buffer; (7) OTA standard solutions, with concentrations of 0, 2.0, 5.0, 10, 20, 50, 100, and 200 pg mL^−1^, were prepared by diluting OTA working solution (1000 ng mL^−1^) with an assay buffer; (8) blocking solution was 1% casein in the assay buffer; (9) substrate solution was prepared by adding 200 μL of 10 mg mL^−1^ TMB dissolved in DMSO, 3.5 μL of 30% H_2_O_2_, and 1 mL of acetate buffer to 20 mL of ultrapure water; and (10) sulfuric acid (5%) was used to stop the enzymatic coloration.

### 3.3. Synthesis of OTA–Protein Conjugates

OTA is a small molecular compound with a molecular weight of 403, which is called hapten because it has insufficient immunogenicity to stimulate an animal to produce antibodies against OTA. However, by coupling with the carrier protein, the formed OTA–protein conjugate can be used as an immunogen to produce antibodies. As OTA contains a carboxylic group, which can react with the amino group in the carrier protein to form an amide bond, in this study, the OTA–protein conjugate was prepared by the activated ester method. Briefly, 2.0 mg of OTA, 2.0 mg of NHS, and 3.0 mg of DCC (molar ratio 1:2:4) were dissolved in 2.0 mL of anhydrous THF. The mixture was stirred and reacted for 24 h at room temperature in the dark. The activated solution was centrifuged at 10,000 g for 15 min. After removing the white precipitate, the supernatant was dried with nitrogen gas to evaporate the THF. The residue was redissolved in 400 μL of DMF, and the activated hapten solution was slowly added to the BSA (or OVA) solution dropwise (15 mg of protein dissolved in 2 mL PBS buffer, pH 7.4). The coupling reaction was carried out at 4 °C for 4 h under slight stirring. The solution was transferred into a dialysis bag and dialyzed in PBS buffer (0.01 mol L−1, pH 7.4) for 3 days, with several changes in the buffer solution. The dialyzed solution was lyophilized to a powder form, and the resulting OTA–BSA (or OTA–OVA) conjugate was stored in a −20 °C refrigerator until use. OTA–OVA was used as the coating antigen for the establishment of an ic-ELISA, while OTA–BSA was used as the immunogen for the production of mAb against OTA. 

### 3.4. Production of Monoclonal Antibody against OTA 

In this study, the mAb against OTA was produced by the hybridoma technique using OTA–BSA as an immunogen. Five female BALB/C mice (6–8 weeks) were immunized with multiple subcutaneous injections of OTA–BSA. Each mouse was inoculated with an immune dose of 100 μg. For the first immunization, the immunogen, dissolved in physiological saline, was emulsified with an equal volume of Freund’s complete adjuvant. Next, three sequential booster immunizations of 100 μg of immunogen emulsified with the same volume of incomplete Freund’s adjuvant was given to each mouse in the same way at 3-week intervals after the initial immunization. Seven days after the fourth immunization, blood was collected from the tail of the immunized mice, and the titer and affinity of the antisera were measured by ic-ELISA using OTA–OVA as the coating antigen. Three days before cell fusion, the mice were given the final booster injection intraperitoneally with 100 μg of immunogen without adjuvant. 

Among the five immunized mice, one mouse, with the highest affinity recognition for OTA, was sacrificed via cervical dislocation. The spleen lymphocytes obtained from that mouse were evenly mixed with myeloma cells (SP2/0) at a 10:1 ratio and fused in 50% polyethylene glycol (PEG) 4000 under aseptic conditions. After cell fusion, the cell suspension was dispersed in 96-well cell culture plates containing RPMI-1640 medium, 20% fetal bovine serum, and 1% HAT (*v*/*v*) and cultured in an incubator at 37 °C with 5% CO_2_. After 10 days of cell culture, the positive hybridoma cells that were able to specifically secrete antibodies against OTA were selected by ic-ELISA using OTA as a competitor and were sub-cloned by the limiting dilution method. The cells from positive pores with high absorbance were diluted and distributed into 24-well cell culture plates. The supernatants were collected and measured by ic-ELISA again for second screening to select cells with a sufficient inhibitory effect. After subcloning, stable mAb-producing clones were expended and cryopreserved in liquid nitrogen. Parts of stable subclones were injected into the abdominal cavity of nude BALB/C mice to generate mAbs. One week later, the ascites from the mice were collected and purified by protein G immuno-affinity extraction. The purified mAb was stored at −20 °C in the presence of 50% glycerol.

Animal welfare and experimental procedures were performed in strict accordance with the recommendations in the National Institutes of Health Guide for the Care and Use of Laboratory Animals (23a). All protocols were approved by the Soochow University Institutional Animal Care and Use Committee (IACUC). All efforts were made to minimize animal suffering and to reduce the number of animals used.

### 3.5. Procedures of ELISA

Coating antigen (OTA–OVA), diluted with coating buffer, was added to a 96-well microplate (200 μL per well) and left at 4 °C overnight. The plate was washed three times with PBST, and the blocking solution (1% casein, 280 μL per well) was added to the plate, which remained at room temperature for 1 h. After washing the plate with PBST three times again, the OTA standard (100 μL per well) and mAb solution (diluted with PBS, 100 μL per well) were successively added to the plate, which was then gently shaken at room temperature for 1 h. The plate was washed three times again, followed by the addition of HRP-GaMIgG solution (diluted in PBS, 200 μL per well), and was incubated at room temperature for 1 h. After washing the plate, the enzymatic substrate solution (200 μL per well) was added. The enzymatic reaction was performed for 15–20 min with light shaking, and then terminated by adding 5% sulfuric acid (80 μL per well). The absorbances in the wells of the plate were measured with the ELISA reader at 450 nm. The standard curve of the ELISA for OTA was constructed by plotting the (B/B_0_) × 100% ~ log C_OTA_, where B and B_0_ were the absorbances of the standard solution and zero concentration, respectively. From the standard curve, the IC_50_ (e.g., the OTA concentration that produced 50% signal inhibition) can be obtained. A lower IC_50_ value indicates a higher sensitivity of the ELISA for OTA. Concentrations of OTA in samples can be calculated according to the standard curve.

### 3.6. Cross-Reactivity Testing

The specificity of the method was evaluated by cross-reactivity (CR) testing. OTA, OTB, OTC, and the other four mycotoxins (Table 2) were selected for CR assay. Standard solutions for CR detection were prepared at concentrations ranging from 0 to 200 pg mL^−1^ for OTA and from 0 to 1000 ng mL^−1^ for the other tested compounds. After performing ic-ELISA procedures, the values of IC_50_ can be obtained from the corresponding standard curves. The CR (%) was calculated using the following equation: CR (%) = (IC_50_ of OTA/IC_50_ of the tested compound) × 100%.

### 3.7. Sample Spiking and Extraction

To obtain accurate and precise values of the ELISA, three types of samples, including corn, wheat, and feed, were collected from local supermarket in Suzhou (China) for the spiking experiment. All samples were ground and then intensively homogenized. The spiking experiment was performed as follows: For wheat and corn samples, 2.0 g of homogenous sample was individually added to a 50 mL plastic centrifuge tube, then 2~20 μL of OTA working solution (1000 ng mL^−1^) was added into the tubes to prepare a final concentration of 1.0~10 ng g^−1^. For feed samples, 50~200 μL of OTA working solution (1000 ng mL^−1^) was added into the tubes containing 2.0 g of homogenous sample to obtain a final concentration of 25~100 ng g^−1^. Then, 4 mL of 60% methanol was added to the spiked sample. The spiked samples were vortexed for 10 min and centrifuged at 2000 rpm for 15 min. Depending on the concentration spiked, the supernatant was diluted 30~3000 times with PBS and then applied directly to the ELISA procedure. For each sample, three separate extractions were performed, and each sample was determined in triplicate. The un-spiked samples were extracted in the same way and used as blanks. Analytical parameters such as precision and recovery (after subtracting the background concentration) were calculated. The recovery of OTA from the spiked samples was calculated as follows: Recovery (%) = [(OTA concentration measured − Blank)/OTA concentration spiked] × 100%.

### 3.8. HPLC-FLD

An HPLC system (ACQUITY, Arc, Waters, Milford, MA, USA) with a fluorescent detector and C_18_ column (150 mm × 4.6 mm; Particle size: 5 μm) was used for OTA detection. The column temperature was 35 °C. The mobile phase consisted of acetonitrile–water–glacial acetic acid (96:102:2, *v*/*v*/*v*). The flow rate was 1.0 mL min^−1^. The injection volume was 50 μL. The fluorescence excitation wavelength (λem) was 333 nm, and emission wavelength (λex) was 460 nm. OTA standard solutions of 0, 0.2, 1, 2, 5, 10, and 20 ng mL^−1^ were used to establish the standard curve, and the results were analyzed by the external standard method.

## 4. Conclusions

An extremely highly sensitive ELISA in pg mL^−1^ level using newly prepared mAb for the detection of OTA in food samples was developed and validated by HPLC-FLD. The IC_50_ and LOD of the ELISA for OTA were 34.8 pg mL^−1^ and 1.5 pg mL^−1^, respectively, which was 1.53~147 times lower than those in other published ELISAs. There was no cross-reactivity of the assay with AFB_1_, ZEN, DON, and T-2. Due to the high similarity of the molecular structures among OTA, OTB, and OTC, the CR values of the assay with OTB and OTC were 96.67% and 22.02%, respectively, indicating that the ELISA was able to evaluate the total ochratoxin levels in food samples. Good recoveries with accepted RSD values of the ELISA for OTA in spiked samples were obtained. There was a good correlation between ELISA (x) and HPLC (y) for the spiked food samples (corn). It has been demonstrated that the ELISA based on a newly prepared mAb was a feasible and ultra-sensitive analytical method for the detection of OTA in food samples.

## Figures and Tables

**Table 1:** Comparison of published ELISAs with our ELISA for OTA detection.

ELISA	Antibody	IC_50 _ (ng mL^−1^)	LOD (ng mL^−1^)	Ref.
ic-ELISA	pAb	5.0	/	[23]
dc-ELISA	mAb	2.0	0.18	[24]
ic-ELISA	mAb	1.7	0.15	[25]
dc-ELISA	mAb	1.2	0.12	[26]
dc-ELISA	pAb	0.9	/	[27]
ic-ELISA	pAb	0.5	/	[28]
ic-ELISA	mAb	0.38	0.07	[29]
ic-ELISA	mAb	0.37	0.08	[30]
dc-ELISA	mAb	0.32	/	[31]
ic-ELISA	mAb	0.2	0.03	[32]
dc-ELISA	mAb	0.08	/	[33]
dc-ELISA	pAb	0.07	/	[34]
ic-ELISA	mAb	0.058	/	[35]
ic-ELISA	mAb	0.052	0.008	[36]
ic-ELISA	mAb	0.0348	0.0015	This work

**Table 2:** Cross-reactivity of ELISA for OTA and the other tested compounds.

Compound	Molecular Structure	IC_50_ (ng mL^−1^)	CR (%)
OTA	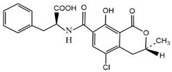	0.0348	100
OTB	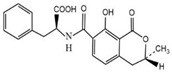	0.0356	96.67
OTC	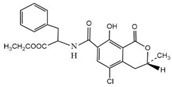	0.158	22.02
AFB_1_	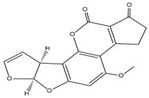	>1000	<0.01
ZEN	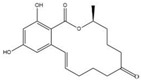	>1000	<0.01
DON	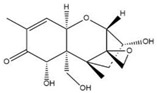	>1000	<0.01
T-2	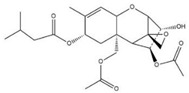	>1000	<0.01

**Table 3:** The recoveries and coefficients of variation of the ELISA for the detection of OTA in the spiked samples.

Sample ^a^	Spiked Volume ^b^ (μL)	Con. Spiked (ng g^−1^)	Dilution Times	Con. Measured ± SD (ng g^−1^)	Recovery (%)	RSD (%, *n* = 3)
Wheat	0	0	30	0.23 ± 0.015	/	6.5
	2	1	30	1.34 ± 0.18	110.8	13.6
	10	5	150	5.01 ± 0.37	95.6	7.3
	20	10	200	10.57 ± 0.55	103.4	5.2
Corn	0	0	30	0.26 ± 0.023	/	8.8
	2	1	30	1.20 ± 0.16	94.4	13.0
	10	5	100	4.89 ± 0.40	92.6	8.2
	20	10	300	9.21 ± 0.98	89.5	10.6
feed	0	0	500	6.30 ± 0.71	/	11.2
	50	25	1000	34.63 ± 2.67	113.3	7.7
	100	50	2000	54.85 ± 7.51	97.1	13.7
	200	100	3000	98.10 ± 12.75	91.8	13.1

^a^ Two grams of samples; ^b^ OTA standard concentration at 1000 ng mL^−1^.

## Data Availability

Not applicable.

## Sample Availability

Food samples are available from supermarkets in Suzhou, China.
